# Understanding how language barriers in the paediatric emergency care setting influences safety of care delivery: a scoping review

**DOI:** 10.1136/emermed-2025-215617

**Published:** 2026-05-04

**Authors:** Ricky Odedra, Phoebe Averill, Ruud Gerard Nijman, Veronika Wiemker, Bhairavi Hariharan, Erik Mayer

**Affiliations:** 1 NIHR North West London Patient Safety Research Collaboration, Imperial College London, London, UK; 2 Better Health & Care Hub, King’s College London, London, UK; 3 Section of Paediatric Infectious Diseases, Imperial College London, London, UK; 4 Department of Paediatric Emergency Medicine, Imperial College Healthcare NHS Trust, London, UK; 5 Section of Health Equity Studies & Migration, University Hospital Heidelberg Department of General Practice and Health Services Research, Heidelberg, Germany; 6 Department of Neonatology, University Hospital Heidelberg Center of Paediatric and Adolescent Medicine, Heidelberg, Germany; 7 iCARE Secure Data Environment & Digital Collaboration Space, NIHR Imperial Biomedical Research Centre, London, UK

**Keywords:** safety, communication, pediatric emergency medicine

## Abstract

**Background:**

Communication in a family’s primary language can support safe care. Vital steps within the care delivery process are contingent on successful communication, including reporting symptoms, clinical decision-making, informed consent, discharge communication and follow-up coordination. The importance of effective information exchange is particularly pronounced in paediatric emergency care, and complex interactions may arise as parents or carers advocate on behalf of children. This scoping review aimed to identify and map existing research indicating where along the care journey communication-related risks for safety lie during paediatric emergency care and what strategies exist to mitigate them.

**Methods:**

We searched MEDLINE, Embase, CINAHL, Scopus, Web of Science and Cochrane Library for studies which examined the influence of language barriers on patient safety in paediatric emergency care as well as studies that evaluated interventions. Bibliographic database searches were executed on 18 December 2024; retrieved records were independently screened by two authors at title and abstract level followed by full text level. Data on study objectives, population characteristics, study design and their key findings were extracted.

**Results:**

1578 articles were identified, of which 33 were included and mapped according to (i) studies reporting safety risks linked to language barriers in paediatric emergency care (n=24) and (ii) existing interventions designed to mitigate these risks (n=9). Studies highlighted that language barriers can influence safety at multiple stages of the emergency care pathway, with discharge most frequently reported as a point of risk for paediatric patient safety. Interventions focused primarily on usage, uptake and documentation of professional interpreter services.

**Conclusion:**

Addressing misunderstandings around follow-up and home-care advice during medical safety netting are priority areas for intervention. Future research should involve carer and clinical perspectives in exploring whether technology-enabled tools, including artificial intelligence, can safely mitigate language barriers in these situations.

**WHAT IS ALREADY KNOWN ON THIS TOPIC**

**WHAT THIS STUDY ADDS**

- This scoping review identified 33 studies, with the discharge process emerging as a high-risk point for safety, while most interventions centred on interpreter service uptake. We found that no studies evaluated digitally-integrated tools or artificial intelligence (AI)-based translation tools.

**HOW THIS STUDY MIGHT AFFECT RESEARCH, PRACTICE OR POLICY**

- Future interventions and quality improvement initiatives should consider key stakeholder perspectives, including those of carers facing language barriers and clinical staff in paediatric emergency care.
- Digital and AI-based tools should be rigorously tested to determine their safety and efficacy before being adopted as solutions to language barriers.

## Introduction

 Clear communication underpins patient safety in paediatric emergency care, for example, when eliciting medical history,^1^ supporting shared decision-making,^2^ obtaining informed consent^3^ and conveying discharge instructions.^4^ This includes clarity of safety netting advice, comprised of guidance on what symptoms to monitor, what to do if new concerns arise or when and how to seek further care.^5^ In multilingual societies, the language(s) spoken by patients and families may not always align with the primary language of care. These language barriers may amplify safety risks. Previous studies have highlighted safety inequities for populations who experience language barriers. This includes poorer healthcare outcomes, medication administration and management of pain.^6–9^

There are distinct pressures during paediatric emergency care. Staff must triage rapidly but accurately across a range of acuity levels using information that is often incomplete or unclear.^10^ Unpredictable surges in patient numbers and service demand impact on the time available for personalised communication with patients. Under these conditions, clinicians may adopt informal strategies such as using ad hoc interpreters for overcoming language barriers in clinical consultations, which can lead to misunderstandings and errors.^11^ The need for rapid decision-making also leaves little room for exploring linguistic or cultural nuances associated with language diversity. Moreover, paediatric emergency care differs from adult services in that many children may not be able to describe symptoms and/or their medical history independently. Clinicians therefore rely on accompanying parents or carers—hereafter termed carers—for key details and consent. This triadic communication exchange makes paediatric consultations particularly complex.

Ad hoc interpreting is sometimes taken on by bilingual staff, family members, friends or even the presenting child.^12^ Using a paediatric patient or a fellow child sibling places them in a vulnerable dual role. Evidence shows that ad hoc interpreters make additions, omissions and substitutions.^13^ Children may also be exposed to sensitive or distressing information, disrupting family roles.^14^ The emotional load can be high, potentially raising safeguarding concerns. For these reasons, the use of professional interpreters is considered the standard to support safe care delivery.

There is a lack of consensus as to how language barriers influence safety in paediatric emergency care. Definitions, outcomes and measures used in existing research vary, limiting comparability and translation into practice. In this review, we conceptualised “patient safety” using Vincent’s definition, as the avoidance, prevention and amelioration of adverse outcomes or injuries arising from healthcare processes.^15^ We interpreted this broadly to encompass both direct safety outcomes (eg, medication or diagnostic errors) and indicators relevant to safety that may signal vulnerabilities in processes along the care journey (eg, self-discharge before medical assessment and delays in care). Furthermore, we operationalise a definition of quality which encompasses safety as a component of quality.^16^ Through a systematic search of the literature, this scoping review aims to identify where along the care journey risks arise, what interventions exist and where the evidence is lacking.

## Methods

### Design

A systematic search focused on language barriers and patient safety in the paediatric emergency care setting. Scoping review methods are well suited as the evidence base in this field remains broad and context-dependent.^17^ JBI guidance for scoping reviews was followed. The review protocol was prospectively registered (osf.io/yt7ck).^18^

### Eligibility criteria

The review conformed to the Preferred Reporting Items for Systematic Reviews and Meta-analyses extension for Scoping Review (PRISMA-ScR). Eligibility criteria aligned with the Population, Concept, and Context (PCC) framework. We included studies involving children (<18 years) and/or their accompanying carers who faced language barriers (Population) and how these barriers relate to patient safety (Concept) in paediatric emergency care settings (Context). The concept domain included both direct patient safety outcomes and safety-relevant indicators of vulnerability along the care journey, where studies linked these to language barriers. Studies were eligible if they examined communication across language barriers within hospital care or at interfaces that directly inform assessment processes. Pre-hospital studies were included only where the results captured information relevant for handover to emergency department triage or assessment. We included published and grey literature reporting on primary research of any design, language or publication year. We excluded work centred exclusively on adult care, non-emergency care, or communication barriers unrelated to spoken or written language (eg, culture or non-verbal communication), as well as studies centred on sign languages.

### Information sources and search strategy

Six bibliographic databases were searched on 18/12/2024 using English search terms: MEDLINE, Embase, CINAHL, Scopus, Web of Science and the Cochrane Library. OpenGrey and Overton were searched for grey literature. Backward citation searching of selected reference lists was conducted as part of handsearching. The full search strategy is presented in online supplemental material 1. Retrieved records were exported to the reference management software Zotero for de-duplication and then imported to the systematic review management platform Covidence for screening.

### Study selection process

Prior to formal screening, eligibility criteria were piloted on a random set of 25 records by three reviewers (RO PA BH). Discrepancies were discussed and refinements were made to clarify approaches to mixed-setting studies and to specify that language barriers had to be a central focus. Formal screening proceeded in two stages. A crossover approach to double-screening was used: one reviewer (RO) screened all records (100%) at both stages while two additional co-authors each screened a proportion of records (20% and 80%, respectively). Two reviewers (RO and PA or BH) independently screened titles and abstracts. Records determined to be relevant were screened at full-text level by two independent reviewers (RO and PA or BH). Exclusion reasons were noted and conflicts resolved through discussion. Two German-language full-text articles were assessed by a native bilingual co-author (VW) and deemed not to meet eligibility criteria.

### Data charting and synthesis

Author, year of publication, country, aim, study design, population, intervention and comparator (if applicable) and key findings were extracted for included articles. Following extraction, studies were mapped to two pre-defined objectives: identifying safety risks linked to language barriers and describing interventions designed to address these risks. Data extraction and charting were piloted on 10 articles. After discussion, adjustments were made to separate study design and population headings and clarification as to what constituted key findings. Charting was conducted by a single author (RO) with colleagues (BH, RGN, PA) verifying consistency throughout. Recurrent themes and evidence gaps were identified within the results.

## Results

**Figure 1:**
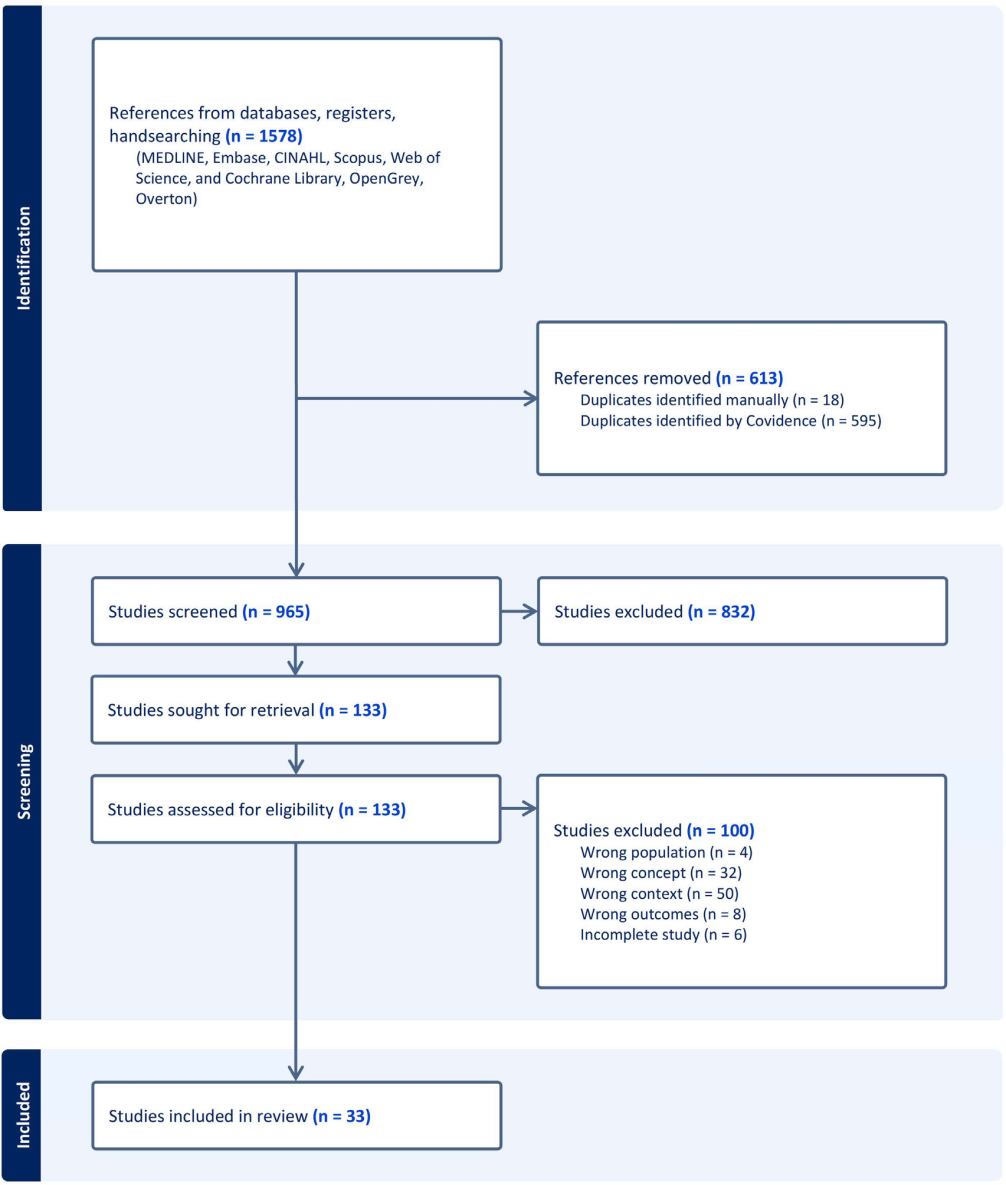
Preferred Reporting Items for Systematic Reviews and Meta-analyses extension for Scoping Review (PRISMA-ScR) flow diagram, created with Covidence (https://www.covidence.org/).

Searches yielded 1578 articles. After removing duplicates, 965 unique titles and abstracts were screened, with 832 records excluded for failing to meet inclusion criteria. Full texts of 133 articles were screened, with 33 studies meeting inclusion criteria for data extraction and charting, among which no grey literature articles were included (figure 1). Almost 80% (n=26) of studies were conducted in the USA, while all other studies were carried out in similarly high-income countries including Switzerland, Canada, Denmark, Germany and Australia. Eligible studies were grouped according to study objectives. One set explored how and where language barriers influence safety along the care journey for patients and carers presenting to paediatric emergency care (n=24) (table 1, online supplemental material 2). Another group centres on existing interventions that address language barriers in paediatric emergency care (n=9) (table 2, online supplemental material 2).

**Table 1:** Summary of where along the care journey communication-related risks for safety lie

Stage in care journey	Description	Studies	Total (n=24)
Registration and triage	Studies relating to language barriers influencing safety at the point of entry to paediatric care.	^20 21^	2
Cross-cutting information exchange	Studies reporting on communication that occurs throughout multiple stages of an emergency care encounter, including history-taking, explanations, consent and shared decision-making and education.	^22–31^	10
Discharge and follow-up	Studies highlighting how language barriers impact discharge and follow-up including return visits, safety netting advice and length of stay.	^32–39 41–44^	12

**Table 2:** Summary of intervention types to address language barriers in paediatric emergency care

Intervention type	Description	Studies	Total (n=9)
Interpreter focused	Interventions involving the use of in-person, telephone, video, interpreter support.	^19 45 46^	3
Visual and picture-based tools	Image-led tools to support history-taking and disease management.	^47 48^	2
Quality improvement and workflow redesign	Process and system changes to improve identification and delivery of language support.	^49–51^	3
Training and simulation	Staff education using simulation methodology.	^52^	1

Across studies examining how and where language barriers influence safety along the care journey for families (table 1), designs were mainly quantitative (n=18/24, 75%), with the largest groups being retrospective cohort studies (n=6) and cross-sectional analyses (n=5); qualitative studies (n=4/24, 17%) and mixed-methods studies (n=2/24, 8%) were less common. Among studies evaluating interventions to address language barriers in paediatric emergency care (table 2), quality improvement approaches were frequent (n=3/9, 33%), followed by randomised trials (n=2/9, 22%) and survey-based evaluations (n=2/9, 22%), with the remainder using a usability testing design (n=1/9, 11%) or interviews (n=1/9, 11%).

Participant representation varied by sample size and participant type in the studies included (table 3, online supplemental material 1). Parents or carers were included in 11 studies (33%) with a median (N) of 48 (IQR 14.5–205). Analyses of index encounters appeared in nine studies (n=63 601; IQR 101–1 19 782), and six studies (18%) reported on families or parent-child dyads (n=297.5; IQR 222.3–984.8). Patients as participants were included in five articles (n=1847; IQR 1430–2590), while healthcare staff samples were reported in only three studies (n=24; IQR 14–30). One study did not report a sample size.^19^ The perspectives of either staff or carers in developing or testing interventions to address language barriers were included in three studies (9%). Carer samples were reported in each of these studies (n=13; IQR 9–51.5), while staff were represented in one (n=4).

### Language barriers and patient safety

#### Registration and triage

At point of entry into paediatric emergency care, evidence suggests that language barriers are poorly documented and are not sufficiently addressed. Interpreter use in emergency departments in Australia was documented in only one of 78 visits spanning 34 patients involving care of patients with limited English proficiency (LEP).^20^ In these encounters, medical procedures were conducted on 22 patients without documented interpreter use. Similarly, analysis of 1430 paediatric emergency cases in Germany found that documentation of patient information such as pre-existing conditions and current medication was lower in cases where language barriers were present compared with German-speaking patients.^21^ These gaps in initial assessments demonstrate how language barriers can compromise completeness of patient clinical information.

#### Cross-cutting information exchange

Studies note the influence of language barriers on clarity and accuracy of information exchanged between families and healthcare professionals, for example, with impacts on carer comprehension of diagnosis.^22 23^ In consults where communication barriers are present, it is difficult to adequately explain medical histories, as shown in a study exploring asylum seekers’ perspectives.^24^ Consistent with this, Latina mothers reported delays or absence of interpreter support in emergency care and often had to ‘get by’ using gestures.^25^ Aronson *et al*^26^ found that clinical decision-making was impeded if families preferred a language other than English, especially when interpreters facilitated communication. In line with this, Spanish-speaking LEP parents were more likely to report concerns with coordination of care received. When professional interpreters were used, parents who perceived the interpretation to be inaccurate were more likely to report problems with information and education relating to their child’s care.^27^ Another study of Spanish-speaking families noted less engagement in discussions about asthma management, while the language barrier reduced their ability to gain education about the disease.^28^ Research also explores implications of language barriers for clinical outcomes. Flores *et al*^29^ analysed 57 professional and ad hoc interpreter-facilitated encounters in a paediatric emergency department over 30 months, identifying 1884 errors. Of these, 18% carried potential clinical ramifications. Their findings also suggest that the presence of a professionally trained interpreter with more than 100 hours of training was associated with fewer clinical errors (12%) compared with ad hoc interpretation (22%) or no interpreter (20%). Further evidence comes from a study reporting that Spanish-speaking families with LEP had greater odds of appendiceal perforation and were less likely to receive early imaging.^30^ Nevertheless, Lowe *et al*^31^ examined diagnostic safety, finding that a language barrier alone was not linked to greater odds of errors.

#### Discharge and follow-up

Language barriers also affect safety downstream at the point of discharge from paediatric emergency care. Gallagher *et al*^32^ reveal that children from LEP homes were significantly more likely to return to emergency care, with readmission within a 72-hour period. Other studies similarly reported higher likelihood of 72-hour revisits for LEP patients.^33 34^ In contrast, Smith *et al*^35^ found no evidence of language barriers as a precondition for return visits. Notably, findings differed by follow-up period as Spanish-speaking families were less likely to return within a year compared with English-speaking families.^34^

Discharge without appropriate safety netting appeared to be prominent for families that may not be proficient in the primary language of care. Gutman *et al*^36^ reported that 31% of discharge interactions occurred without an interpreter present. They also found that only 70% of patient visits resulted in complete discharge education, with the use of professional interpreters positively associated with families receiving full instructions. This included guidance on medication and safety netting advice. Contrary to Gutman *et al* findings, Zamor *et al*^37^ found gaps in understanding of a child’s diagnosis and the safety netting advice provided, regardless of whether an interpreter was used. Riera *et al*^38^ also noted LEP carers had lower usage of an asthma action plan, with language proficiency the only demographic factor associated with plan use. When controlling for income, health literacy and discharge language, Spanish-speaking parents remained more likely to make dosing errors following discharge from emergency care.^39^

Lengthy stays in the emergency department have been associated with poorer outcomes.^40^ In a study assessing children whose parents faced language barriers, the authors reported that these children had longer emergency department visits.^41^ Olivarez *et al*^42^ found that Spanish-speaking families experienced prolonged delays in time from discharge decision-making to actual discharge initiation, whereby overall wait times to discharge increased despite implementation of an electronic medical record system. Meanwhile, waiting times decreased for English-speaking families. Contrastingly, a study evaluating paediatric patients with LEP found that their visits were shorter compared with those not facing language barriers.^43^ Another concluded that, aside from encounters with American Indians, patients using an interpreter or that spoke a language other than English had lower likelihood of leaving without complete evaluation and treatment by a physician.^44^

### Interventions to mitigate language barriers

#### Interpreter-focused

Three studies evaluated the comparative effectiveness of different interpreter modalities in paediatric emergency care.^19 45 46^ In one study, Plan-Do-Study-Act cycles were used to increase telephone interpreter use by introducing multiple components including scripts, electronic medical record flagging and staff education.^19^ Although documentation of interpreter use or non-use increased from 10% to 76%, actual utilisation of telephone interpreters remained low (18.6%). This indicates persistent implementation barriers beyond identifying language needs alone. Crossman *et al*^45^ conducted a prospective, randomised, semi-blinded trial comparing telephone interpretation, in-person interpretation and consultations with a bilingual physician. Patient-provider agreement was consistently high across telephone interpretation, in-person interpretation and bilingual physician consultations, with no statistically significant differences between groups. They conclude that telephonic and in-person interpreters were demonstrated to be non-inferior to bilingual physicians. In assessing video and telephone interpreters, a study found that video interpretation—although more costly—better supported parents’ comprehension of their child’s diagnosis.^46^

#### Visual and picture-based tools

Two studies examined the use of visual communication tools as aids to support overcoming language barriers. One developed an image-based digital tool which was overall positively received by paediatric nurses and migrant parents during usability testing.^47^ Both paediatric nurses and migrant parents reported the tool to be helpful for initial triage and obtaining basic medical history-taking. However, there were difficulties in conveying timeframes and dosages for medication using images alone. Nurses emphasised early use in interactions rather than solely as a fallback strategy. In a survey study, Pade *et al*^48^ evaluated parental perceptions of picture-based medication plans for asthma management. For survey items assessing potential intervention benefits, between 67% and 88% of parents agreed or strongly agreed with its use.

#### Quality improvement and workflow redesign

Three studies investigated workflow redesign and quality improvement methods, aiming to enhance interpreter service usage during paediatric emergency care.^49–51^ One study introduced additional features to existing electronic health record-based workflows. Gupta *et al*^49^ implemented screening questionnaires at triage alongside icons and alerts to encourage identification of language support needs and increase interpreter uptake. This yielded improvements in identification (from 60% to 77%), interpreter service usage (77% to 86%) and documentation rates (38% to 73%). Similar implementation was trialled in a study that used tracking icons for LEP patients and standardised documentation forms.^50^ Interpreter use and documentation both improved but there was no significant reduction in 48-hour return to the paediatric emergency department. While these quality improvement efforts successfully improved practices around documentation of interpreter needs, they suggest that deeper systemic changes are necessary to convert this into improved patient outcomes.^51^

#### Training and simulation

Only one study assessed the role of training and simulation in addressing language barriers. Hendry *et al*^52^ delivered a simulation module to thirty-three emergency medicine residents and three paediatric emergency medicine fellows in the USA. Prior to training, most participants reported limited awareness of institutional and federal language barrier policies. Participants reported that prior to the module, they used family and staff to interpret conversations, and 92% did not use telephone or professional interpretation services. The main reported barrier was time constraints in the emergency department. Following the simulation training, all participants reported improved understanding of managing language barriers, existing interpreter regulations and organisational policies. Most (94%) indicated that the training would change their clinical practice, while 82% recommended the training to become compulsory.

## Discussion

This scoping review systematically synthesised evidence across 33 studies for how language barriers impact safety in paediatric emergency care, alongside identified interventions designed to address those risks. Findings highlight that safety risks occur at multiple points along the care pathway, from registration and triage^20 21^ to discharge and follow-up.^27 29 30 32 33 37–39 42^ Interpreter services, while available, are inconsistently used and overall, poorly documented.^20 32 37^ Interventions largely targeted the uptake of professional interpreter services in various modalities or included adjustments to the design of workflows to encourage interpreter use for language support.^19 45 49 50^ However, these efforts did not consistently demonstrate improvements in clinical outcomes for patients.

These findings show shortfalls in how language barriers are managed during paediatric emergency care. Early lapses in flagging language needs, during registration or triage, have knock-on effects downstream, resulting in incomplete histories, delays in diagnosis and errors.^20 21 30^ Even when interpreters are present, there is reported misunderstanding of discharge instructions or follow-up plans, suggesting that interpreter use alone is insufficient to close comprehension gaps.^27 37 39^

Only three articles that studied interventions explored the perspectives of carers or healthcare staff involved in communication amid language barriers.^47 48 51^ Carers are an important stakeholder as they are typically responsible for communicating on behalf of a paediatric patient, while staff perspectives may contribute to understanding barriers and facilitators of interventions. Despite this, their participation in informing interventions is markedly low. This gap limits insight into why existing supports are not used as intended, while hindering the development of workflow-appropriate interventions. Future studies incorporating carer and clinician perspectives on language barriers could help to identify more deep-rooted barriers to using language supports, such as systematic bias and discrimination, as has been described in other medical settings.^53^ It may also reveal how acceptable tools are from a carer’s perspective of safety.^54^

There is a need to treat language barriers and respective support as priorities for upholding patient safety, rather than a supplementary service. Systematic identification of language preference has at times been successfully embedded into routine workflows, but few studies track cohorts from first contact in an emergency department through to discharge. Future research should identify how miscommunication in early stages of the care pathway can cascade or result in cumulative harm. There is also a clear need for interventions that are informed by families and frontline healthcare professionals, as participatory approaches were rarely employed in intervention design within identified studies. Such approaches can inform interventions that are safety-centred yet culturally sensitive. For policy, interpreter provision should be recognised as a safety metric.

A notable evidence gap concerns evaluations of digital or artificial intelligence (AI) enabled tools for interpreting or translation, despite increasing policy momentum around their use.^55 56^ Technology-based interventions are small-scale, with prototypes that sit outside of clinical systems. Recent initiatives in this field have progressed and emphasise a path towards automation. While professional interpreters are widely advised,^56 57^ the evolving technical landscape suggests that the safety and usability of existing digital tools should be carefully tested. Research should therefore examine whether these tools can be integrated into paediatric emergency workflows, with appropriate governance to safeguard patient safety.

Included studies were clustered in high-income countries, particularly in the USA, with no eligible work originating from low- and middle-income settings. Consequently, evidence centres on Spanish-speaking families—the second most populous language within the USA. This leaves important populations unrepresented. The absence of studies from several linguistically rich contexts highlights a critical evidence gap, with the UK representing one key example.

### Limitations

This review, consistent with JBI guidance for scoping reviews, conducted no formal appraisal of study quality. While this approach is appropriate for the purpose of mapping evidence, we did not assess the strength or reliability of individual findings. Additionally, although the search strategy was broad and included grey literature, we used only English terms and potentially relevant reports from an international context may not have been included. Finally, authors of included studies were not contacted to clarify unclear or missing information.

## Conclusion

The published evidence indicates that for families facing language barriers, the discharge process in paediatric emergency care is especially susceptible to safety risks. In particular, misunderstandings around medication instructions, including dosing and around follow-ups or safety netting advice represent critical areas for improvement. This highlights the need for future research and quality improvement initiatives that specifically strengthen communication at the point of discharge. Given wider shifts in health systems towards digital innovation, further work should examine whether technology-enabled tools can safely support translation and interpreting for these purposes. Ensuring that such tools are evaluated for safety and accuracy will be essential before they can be integrated into clinical practice.

## Supplementary material

10.1136/emermed-2025-215617 online supplemental file 1

10.1136/emermed-2025-215617 online supplemental file 2

## Data Availability

No data are available.
