# Supplementary material for: Understanding how language barriers in the paediatric emergency care setting influences safety of care delivery: a scoping review
Source: Emerg Med J. 2026 May 4;43(8):e215617. doi: 10.1136/emermed-2025-215617 (PMC13479225; doi:10.1136/emermed-2025-215617)
Supplement: Supplementary file 1 [file emermed-43-8-s001.pdf]

Table 1 – Full search strategy

Dimension	Subject headings	Search keywords/boolean operators
**Population**<br>Paediatric patients/accompanying adult(s) with limited language proficiency in the language of care	**Medline:** Communication Barriers/, Limited English Proficiency/, caregiver/<br>**Embase:** exp. Pediatrics/, exp. communication barrier/, exp. limited English proficiency/<br>**Cochrane:** exp. Communication Barriers<br>**CINAHL:** Infant, High Risk**/,** exp. Communication Barriers/, English	(p?ediatric* OR child* OR adolescen* OR "infan*" OR "neonat*")<br>***AND***<br>("parent*" OR "caregiver*" OR "carer*")<br>***AND***<br>("Limited English Proficiency" OR "LEP" OR "language adj3 barrier*" OR "communication barrier*" OR OR "language discordan*" OR "non-English adj3 speak*" OR "limited English*" OR "primary language" OR "English adj3 proficiency" OR "language
**Concept**<br>Safety of care	**Medline:** Patient Harm/, Patient Safety/, Quality of Health Care/<br>**Embase:** exp. patient harm/, exp. patient risk/, exp patient safety indicator/, exp. health care quality/<br>**Cochrane:** exp. Patient Safety/, exp. Patient Harm<br>**CINAHL:** exp. Adverse Health Care Event/, exp Quality of Health Care/, exp Health Care Errors/	"patient safety" OR "adverse adj3 event*" OR "medical adj3 error*" OR "risk*" OR "safety adj3 incident" OR "Diagnos* error*" OR "quality of care" OR "uncertaint*" OR "error*" OR "harm*" OR "safety manag*" OR "inciden*" OR "patient harm*" OR "sentinel event*" OR "risk manage*" OR "prevent* adj3 harm*" OR "care adj3 quality"
**Context**<br>Emergency care	**Medline: exp.** Emergency Service, Hospital/, Pediatric Emergency Medicine/ exp. Hospitals, Pediatric, exp. Emergency medicine<br>**Embase:** exp. emergency medicine/, exp. emergency ward/<br>**Cochrane:** exp. Emergency Service, Hospital/, Emergency Medicine<br>**CINAHL:** exp. Emergencies/, Emergency Medicine/, exp. Pediatrics/, Hospitals, Pediatric/	emergency medicine" OR "emergency department*" OR "ED*" OR "emergency care" OR "ambulatory" OR "A&E*" OR "p?ediatric* emergency" OR "accident and emergency" OR "tertiary" OR "trauma cent*" OR "emergency service*" OR "urgent care"

Table 2 – Number of articles extracted from databases searched

Database	Search results
Medline	213
Embase	477
CINAHL	375
Scopus	406
Web of Science	74
Cochrane Library	33
**Total (including duplicates)**	**1578**

Table 3 - Descriptive statistics of participant type in included studies

Participant/unit type	Reported	% of 33 studies (reported)*	Median	Q1	Q3	IQR	Min	Max
Staff	3	0.090	24	14	30	16	4	36
Parent/carer	11	0.333	48	14.5	205	190.5	5	630
Children/patients	5	0.151	1847	1430	2590	1160	107	3551
Index encounters	9	0.272	63601	101	119782	119681	57	232787
Parent/child dyads/families	6	0.181	297.5	222.25	984.75	762.5	55	8068
Unreported	1	0.030	-	-	-	-	-	-

* Participant/unit type categories were not mutually exclusive; some studies included more than one group
