# Supplementary material for: Understanding how language barriers in the paediatric emergency care setting influences safety of care delivery: a scoping review
Source: Emerg Med J. 2026 May 4;43(8):e215617. doi: 10.1136/emermed-2025-215617 (PMC13479225; doi:10.1136/emermed-2025-215617)
Supplement: Supplementary file 2 [file emermed-43-8-s002.pdf]

Table 1 Studies included exploring where language barriers influence safety along the care journey in paediatric emergency care

Author, year, country	Objective(s)	Study design	Population	Relevant key findings
Aronson, 2022, USA [26]	To investigate clinicians' and parents' perspectives on decision-making regarding hospitalisation for children with bronchiolitis.	Qualitative: semi-structured interviews	17 physicians, 7 nurses, and 20 parents	Preferred language other than English was identified as a barrier to shared decision making by clinicians, especially when communication depended on interpreters.
Arthur, 2015, USA [27]	To compare care experiences between LEP and English-proficient parents, and to assess interpretation accuracy.	Quantitative: prospective observational study	630 LEP/non-LEP parents	Among LEP parents who received interpretation, those perceiving inaccuracies were over twice as likely to report problems with information/education.
Brandenberger, 2019, Switzerland [24]	To explore asylum-seeking carers' perspectives on care in an emergency department.	Qualitative: semi-structured interviews	13 asylum-seeking carers	Communication barriers caused anxiety and uncertainty, especially during emergency care.
Brophy-Williams, 2020, Australia [20]	To understand the context of interpreter use for Refugee Health Service patients.	Quantitative: retrospective audit	78 ED encounters	Only one Refugee patient out of 78 encounters was provided an interpreter.<br><br>Procedures took place without documented interpreter use in 22 patients spanning 78 encounters.
Claudio, 2009, USA [28]	To determine whether language barriers affect asthma management among Latino children	Quantitative: cross-sectional survey	1847 children attending public elementary schools	Language barriers contributed to lower asthma education and higher acute care utilisation.

LEP: limited English proficiency, ED: emergency department

Table 1 Continued

Author, year, country	Objective(s)	Study design	Population	Relevant key findings
Dahl, 2015, USA [22]	To assess parent/guardian recall of informed consent information provided during emergency procedures and explore how preferred language influences understanding.	Quantitative: surveys	55 parent/child dyads	Language was a reported barrier to obtaining informed consent.
Dungu, 2019, Denmark [23]	To describe the use of professional interpretation in paediatric EDs, and to explore whether language barriers affect clinical management.	Mixed methods: prospective descriptive study	230 patients with Danish speaking parents, 136 non-native-Danish speaking	Clinical management was reported to be affected in 44% of non-native language consults, including prolonged consultations, diagnostic uncertainty, and difficulty conveying instructions.
Flores, 2012, USA [29]	To compare interpreter errors and their clinical consequences across three groups: professional, ad hoc, and no interpreter.	Quantitative: cross-sectional analysis	57 consults with LEP families	1,884 interpreter errors were identified over 57 encounters.<br><br>Professional interpreters had fewer errors of clinical consequence (12%) than ad hoc (22%) or no interpreters (20%).
Gallagher, 2013, USA [32]	To compare the rate of ED return visits requiring admission between children from English-speaking households and those from households with LEP.	Quantitative: retrospective cohort study	119,782 LEP/non-LEP patients	Children from LEP households had higher odds of return admission within 72 hours (1.6% vs 1.2%; OR 1.30, 95% CI 1.12–1.50).
Goldman, 2006, USA [33]	To determine if children whose parents speak a primary language other than English spend longer in the ED.	Quantitative: retrospective cohort study	8,068 families	After controlling for triage acuity, age, and gender, non-English speakers spent longer in the ED.

ED: emergency department, LEP: limited English proficiency, OR: odds ratio, CI: confidence interval

Table 1 Continued

Author, year, country	Objective(s)	Study design	Population	Relevant key findings
Greenky, 2019, USA [34]	To evaluate care of LEP patients.	Quantitative: retrospective cohort study	232,787 encounters	LEP patients showed higher odds of change in triage status, hospital admission, and readmission within 7 days.<br><br>Time spent in emergency care was shorter for LEP patients.
Gutman, 2018, Canada [35]	To assess whether professional interpreter use is associated with improved discharge education.	Mixed methods: video transcript analysis	101 consults from 47 LEP family visits	31% of discharge interactions occurred without an interpreter present.<br><br>Professional interpreter use increased the odds of complete discharge education (OR 7.1, 95% CI 1.4–37.0.
Harrison, 2012, USA [36]	To analyse the influence of race, language, and insurance status on leaving the paediatric ED without being seen by a physician.	Quantitative: retrospective case-cohort study	76,931 encounters	Speaking a language other than English at home was associated with lower odds of leaving without complete evaluation and treatment by a physician.
Levas, 2014, USA [30]	To determine whether ethnicity and LEP are associated with higher rates of appendiceal perforation and reduced use of early imaging.	Quantitative: secondary analysis of a prospective cross-sectional study	2590 patients with suspected appendicitis	Hispanic children with LEP had higher odds of appendiceal perforation and were less likely to receive advanced imaging.
Lowe, 2023, USA [31]	To investigate the relationship between carer preferred language and the occurrence of diagnostic errors.	Quantitative: retrospective cohort study	3551 patients who were admitted within 10 days of an index visit.	Language barriers did not predict higher odds of diagnostic errors.

LEP: limited English proficiency, OR: odds ratio, CI: confidence interval, ED: emergency department

Table 1 Continued

Author, year, country	Objective(s)	Study design	Population	Relevant key findings
Müller, 2023, Germany [21]	To determine whether essential patient information can be obtained from children and carers with/without language barriers during paediatric pre-hospital emergencies.	Quantitative: retrospective cross-sectional study	1430 pre-hospital patients	Documentation of information (pre-existing conditions, current medication, and prior events leading to the emergency) was poorer in cases involving language barriers.
Olivarez, 2017, USA [37]	To understand the process and differences in patient discharge from EDs according to language.	Quantitative: cross sectional observation study	210 English/Spanish speaking families	No differences in verbal interaction or discharge dialogue content.<br><br>Spanish-speaking families had longer wait times from discharge decision to initiation of the discharge process.
Portillo, 2021, USA [38]	To examine the association of LEP with 72-hour paediatric ED revisits.	Quantitative; retrospective cross-sectional and nested case–control analysis	63,601 encounters	LEP was associated with higher odds of 72-hour ED revisits.<br><br>Interpreter use was inconsistently documented and not associated with reduced revisit risk.
Riera, 2014, USA [39]	To examine the impact of LEP versus English proficiency on asthma action plan use.	Quantitative: cross-sectional survey	107 carers of patients presenting with asthma symptoms	Asthma action plan use was lower among LEP carers.<br><br>English proficiency was associated with increased action plan use.
Samuels-Kalow, 2013, USA [40]	To examine the relationship between parental language proficiency and discharge comprehension regarding medication dosing.	Quantitative: prospective observational study	259 English/Spanish speaking carer-children dyads	Spanish-speaking parents were more likely to commit a dosing error than English-speaking parents.

ED: emergency department, LEP: limited English proficiency

Table 1 Continued

Author, year, country	Objective(s)	Study design	Population	Relevant key findings
Samuels-Kalow, 2017, USA [41]	To analyse the relationship between parental language and 72-hour return visits.	Quantitative: secondary analysis of a prospective cohort.	202 English/Spanish speaking carers	Spanish-speaking parents were more likely to return to the ED within 72 hours.<br><br>Spanish-speaking parents had lower odds of returning for a second visit within a year.
Smith, 2022, USA [42]	To evaluate motives for paediatric ED return visits within 72 hours and to evaluate whether these reasons differ by Hispanic ethnicity or primary language.	Quantitative: prospective survey study	499 carers returning to emergency care within 72-hours	No differences in reasons for return by ethnicity or language.<br><br>Spanish-speaking carers more likely to have received verbal instructions only.
Steinberg, 2016, USA [25]	To explore Latina mothers' perspectives on managing language barriers in paediatric healthcare settings.	Qualitative: secondary thematic analysis	48 Latina mothers	Interpretation delays or absence were common in EDs.<br><br>Mothers often relied on gestures or partial understanding to “get by”.
Zamor, 2022, USA [43]	To explore the lived experiences of Latinx families with low English proficiency in paediatric EDs.	Qualitative: phenomenology-based thematic analysis	16 Latinx parents of children with respiratory illnesses	Fear and limited confidence in managing child’s illness at home.<br><br>Gaps in understanding diagnosis and safety netting advice.

ED: emergency department, LOS: length-of-stay, LEP: limited English proficiency

Table 2 Studies assessing interventions aimed at addressing language barriers in paediatric emergency care

Author, year, country	Objective(s)	Study design	Population	Intervention type	Relevant key findings
Bravo, 2024, Canada [19]	To improve language concordant care in a paediatric ED by increasing the rate of over-the-phone interpreter use.	Quality improvement study	Paediatric ED patients/carers with non-English language preference	Interpreter focused	Documentation of interpreter use or non-use in patients increased from 10% to 76%.<br><br>18.6% of patients/carers with LEP had interpreter service use recorded.
Crossman, 2010, USA [45]	To compare the efficacy of telephonic and in-person interpretation against bilingual provider communication in paediatric EDs	Prospective, randomised controlled trial	1201 Spanish speaking LEP families	Interpreter focused	Agreement between parent-reported/recorded discharge diagnoses was similar.
Denecke, 2022, Switzerland [47]	To test a non-verbal, image-based digital communication tool to support paediatric emergency nurses in caring for migrant patients and families.	Usability study	10 participants	Visual and picture based	Nurses and parents found the tool helpful for triage and anamnesis.<br><br>Difficulty in expressing time and quantities.
Gupta, 2023, USA [49]	To improve early identification of LEP and respective interpreter use and documentation.	Quality improvement study	54,922 encounters f	Quality improvement/work flow redesign	LEP identification at triage increased from 60% to 77%.<br><br>Interpreter use among those identified increased from 77% to 86%.
Hendry, 2012, USA [52]	To evaluate the impact of a simulation-based educational intervention on residents' and fellows' knowledge of language barriers and regulations.	Pre- and post-intervention surveys	33 residents and 3 paediatric emergency fellows	Training and simulation	Post-intervention, 100% reported improved knowledge of LEP needs, regulations, and policies.<br><br>94% said it would change their practice.

ED: emergency department, LEP: limited English proficiency

Table 2 Continued

Author, year, country	Objective(s)	Study design	Population	Intervention type	Relevant key findings
Lion, 2015, USA [46]	To determine the effect of telephone interpreting versus video interpreting on parent comprehension of diagnosis.	Randomised clinical trial	208 Spanish-speaking LEP parents	Interpreter-focused	Parents in the video group were more likely than those receiving telephone interpretation to correctly name their child's diagnosis (74.6% vs 59.8%).<br><br>No differences in time spent in emergency care.
Martinez, 2021, USA [50]	To increase appropriate interpreter use and documentation and reduce 48-hour return visit rates	Quality improvement study	154,067 LEP/non-LEP encounters	Quality improvement/work flow redesign	Interpreter use and documentation improved from 35.7% to 64.5%<br><br>48-hour return visit rates remained unchanged.
Pade, 2021, USA [48]	To assess a picture-based medication plan for asthma management	Quantitative: prospective survey study	90 English/Spanish-speaking parents	Visual and picture-based tool	67–88% of parents agreed /strongly supported the tool.<br><br>Helpful in improving understanding of asthma medications and confidence in managing asthma.
Santos Malavé, 2019, USA [51]	To evaluate carers perceptions, understanding, and barriers of an asthma care implementation bundle	Qualitative: semi-structured interviews	Spanish-speaking carers of 492 patients presenting with asthma	Quality improvement/work flow redesign	Carers were uncertain about child's diagnosis and acuity.

LEP: limited English proficiency
